# Supplementary material for: Huntingtin Decreases Susceptibility to a Spontaneous Seizure Disorder in FVN/B Mice
Source: Aging Dis. 2023 Dec 1;14(6):2249–66. doi: 10.14336/AD.2023.0423 (PMC10676795; doi:10.14336/AD.2023.0423)
Supplement: Supplementary file 1 — The Supplementary data can be found online at: www.aginganddisease.org/EN/10.14336/AD.2023.0423. [file AD-14-6-2249-s.pdf]

SUPPLEMENTARY DATA

Huntingtin Decreases Susceptibility to a Spontaneous Seizure Disorder in FVN/B Mice

Jeremy M. Van Raamsdonk[1,2,3,4*], Hilal H. Al-Shekaili[1], Laura Wagner[1], Tim W Bredy[1,5], Laura Chan[1], Jacqueline Pearson[1], Claudia Schwab[1], Zoe Murphy[1], Rebecca S. Devon[1], Ge Lu[1], Michael S. Kobor[1], Michael R. Hayden[1], Blair R. Leavitt[1*]

© 2023. Van Raamsdonk JM et al. Published online at http://www.aginganddisease.org/EN/10.14336/AD.2023.0423

SUPPLEMENTARY DATA

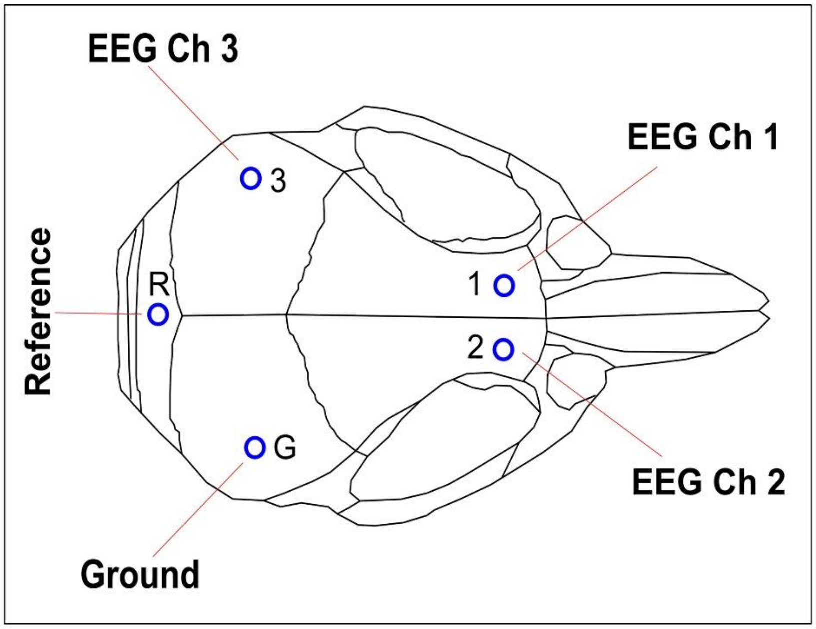

Supplementary Figure 1. Electroencephalogram (EEG) electrode layout. Schematic of mouse cranium showing types and locations of implanted EEG electrodes. Ch = channel.

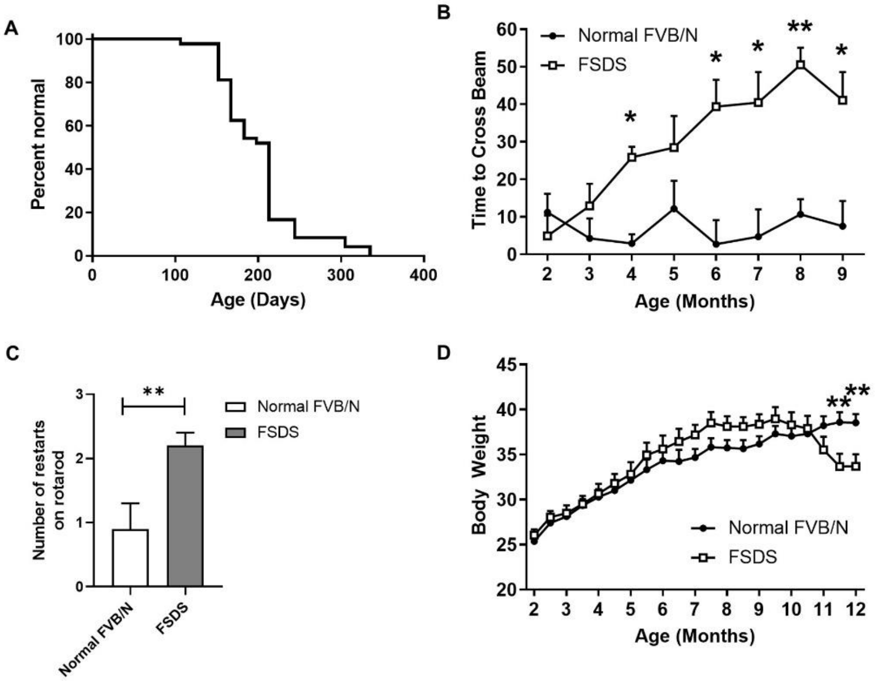

Supplementary Figure 2. FSDS mice show alterations in body weight and abnormal behaviour. To gain further insight into the phenotype of FSDS mice we examined the onset of symptoms, body weight and behaviour **(A)** The onset of the FSDS phenotype occurred between 3.5 and 11 months with an average onset of 6.5 months. **(B)** In a beam crossing test, FSDS mice took significantly longer to cross the beam beginning at about 4 months of age primarily because they were slow to initiate movement. **(C)** FSDS mice also had difficulties in staying on the rotarod primarily due to a refusal to participate in this motor task. **(D)** Phenotypic onset was followed by a period of increased weight which preceded a dramatic drop in weight of 10-15 grams. Error bars indicate standard error of the mean. * $p < 0.05$, ** $p < 0.01$, *** $p < 0.001$.

© 2023. Van Raamsdonk JM et al. Published online at http://www.aginganddisease.org/EN/10.14336/AD.2023.0423

SUPPLEMENTARY DATA

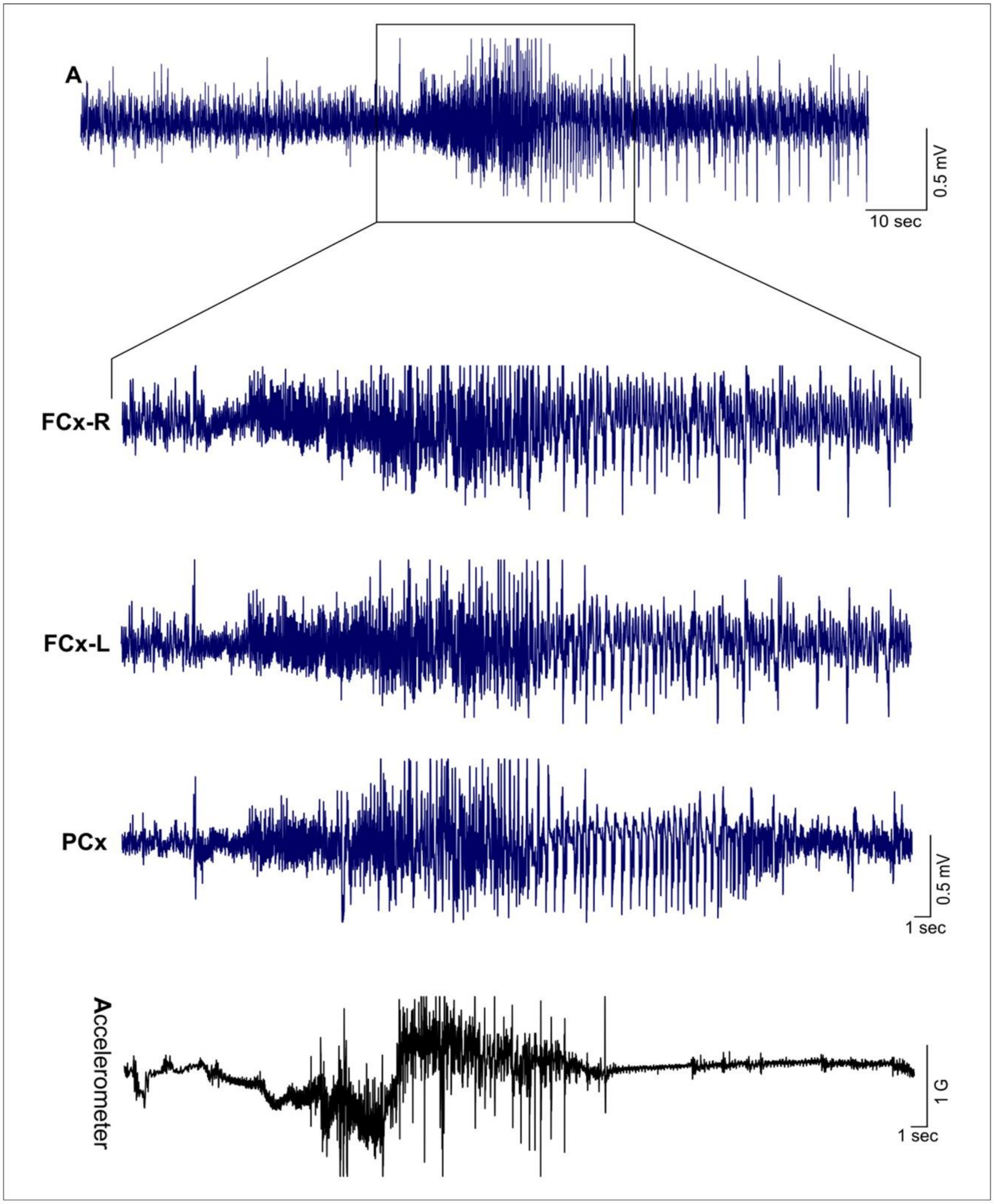

Supplementary Figure 3. Electroencephalogram (EEG) recordings from FSDS mice show epileptiform discharges. Figure S3 and S4 show EEG recordings from two FSDS mice. The top tracing displays a 130-second epoch showing a burst of high-amplitude spike discharges. Underneath is a close-up view of the spike burst showing synchronous epileptiform discharges from the 3 EEG channels along with concurrent trace from accelerometer x-axis. Each trace is 50 seconds in duration. Abbreviations, FCx-L: left frontal cortex, FCx-R: right frontal cortex, PCx: parietal cortex, sec: seconds, mV: millivolts, G: acceleration of Earth's gravity (~9.8 m/s).

© 2023. Van Raamsdonk JM et al. Published online at http://www.aginganddisease.org/EN/10.14336/AD.2023.0423

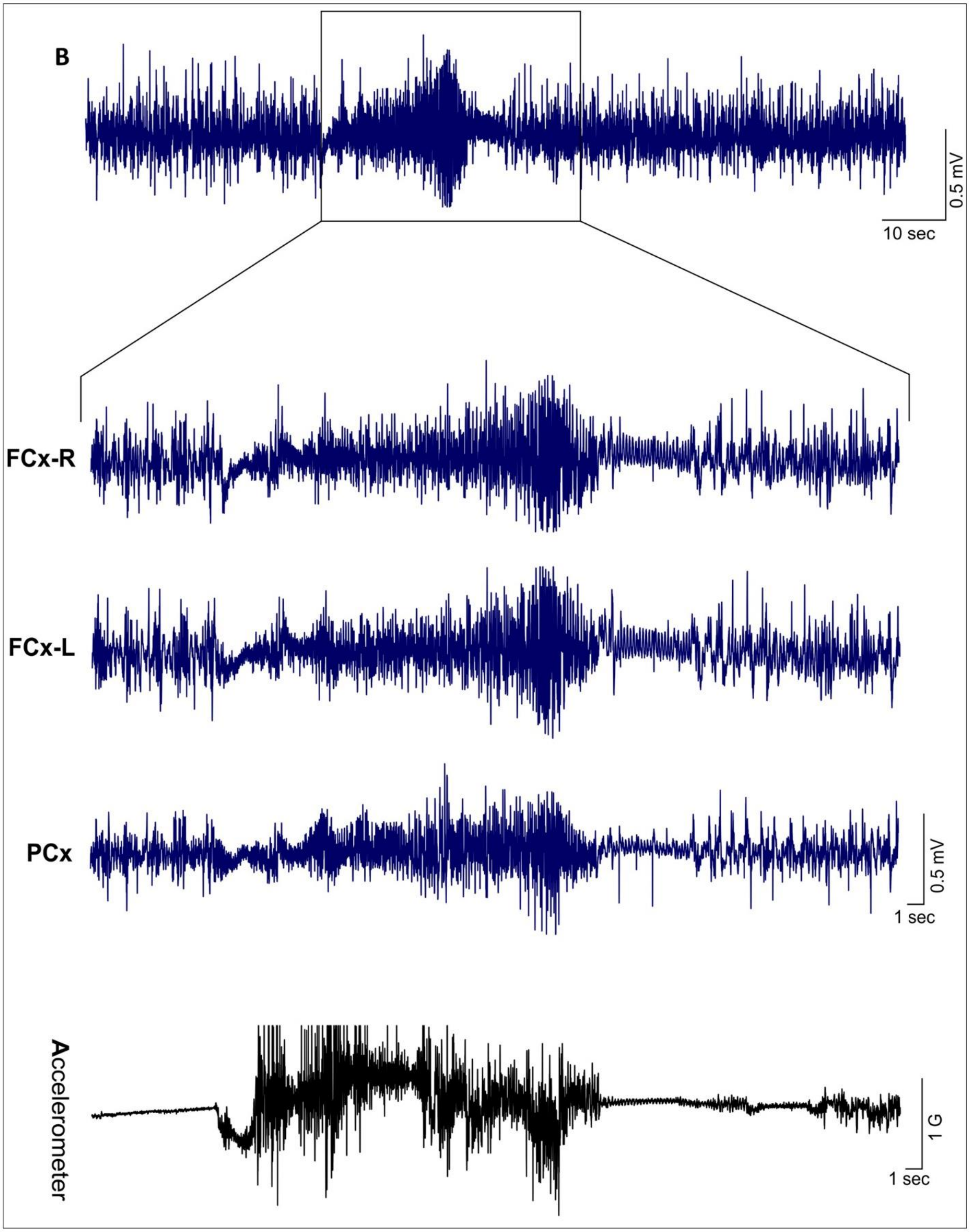

Supplementary Figure 4. Example 2 of epileptiform discharges detected in FSDS mice.

© 2023. Van Raamsdonk JM et al. Published online at http://www.aginganddisease.org/EN/10.14336/AD.2023.0423

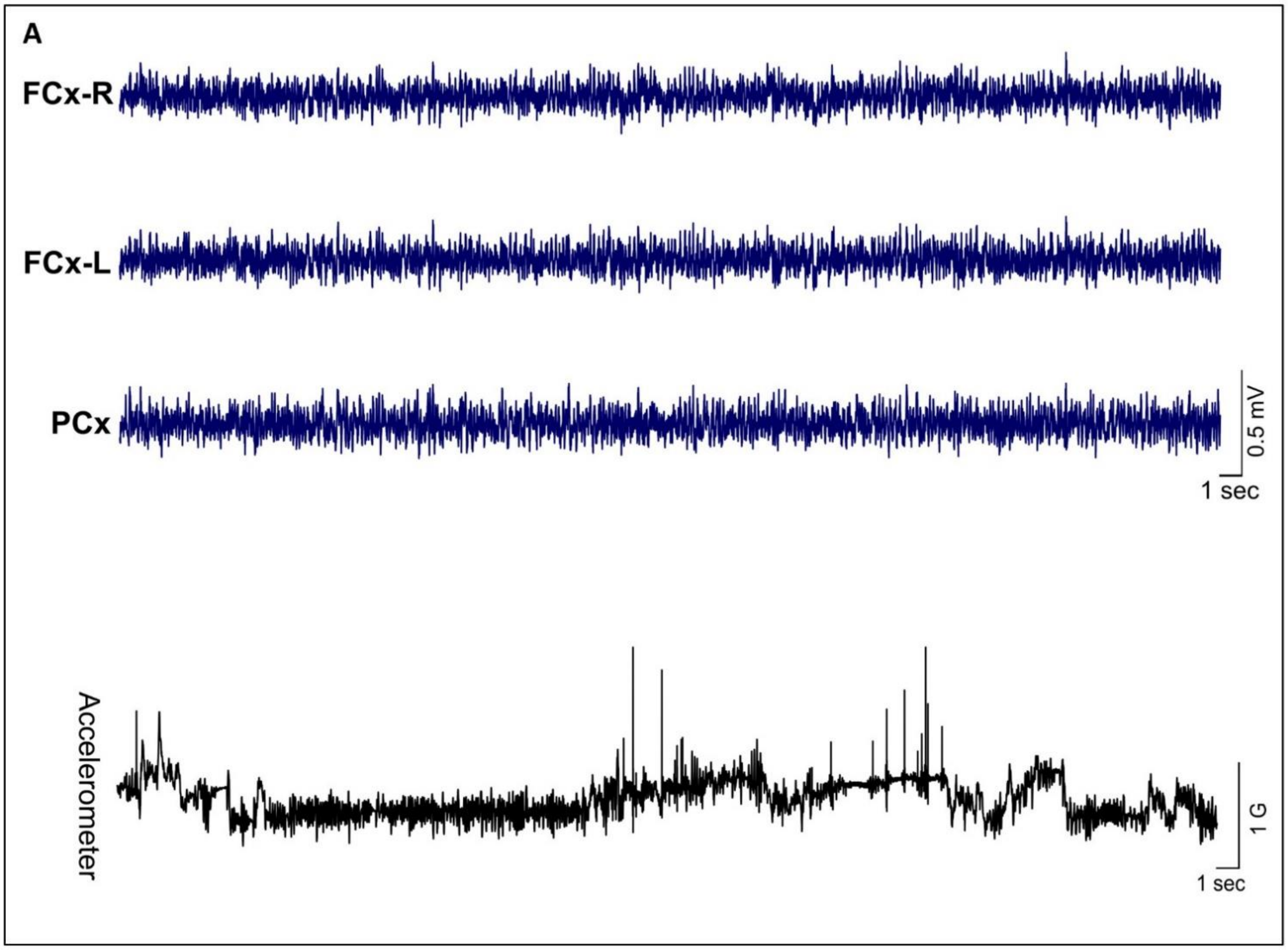

Supplementary Figure 5. Electroencephalogram (EEG) traces from normal WT FVB/N mice do not show any seizure activity – Example 1. Figure S5-S10 represent recordings obtained from one of the six normal FVB/N mice included in this analysis. Each trace is 50 seconds in duration. Abbreviations, FCx-L: left frontal cortex, FCx-R: right frontal cortex, PCx: parietal cortex, sec: seconds, mV: millivolts, G: acceleration of Earth's gravity (~9.8 m/s).

© 2023. Van Raamsdonk JM et al. Published online at http://www.aginganddisease.org/EN/10.14336/AD.2023.0423

SUPPLEMENTARY DATA

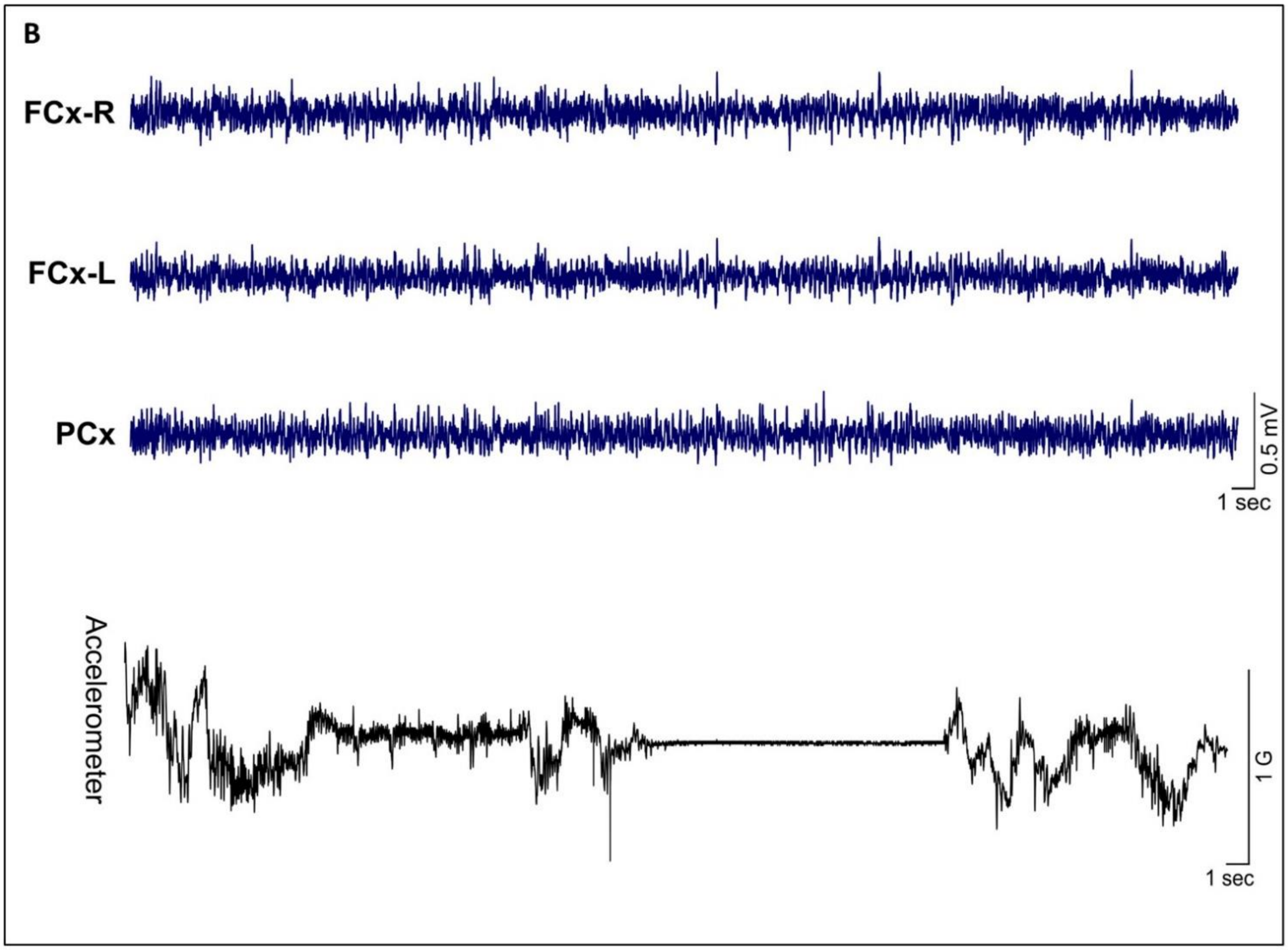

Supplementary Figure 6. Electroencephalogram (EEG) traces from normal WT FVB/N mice do not show any seizure activity – Example 2.

© 2023. Van Raamsdonk JM et al. Published online at http://www.aginganddisease.org/EN/10.14336/AD.2023.0423

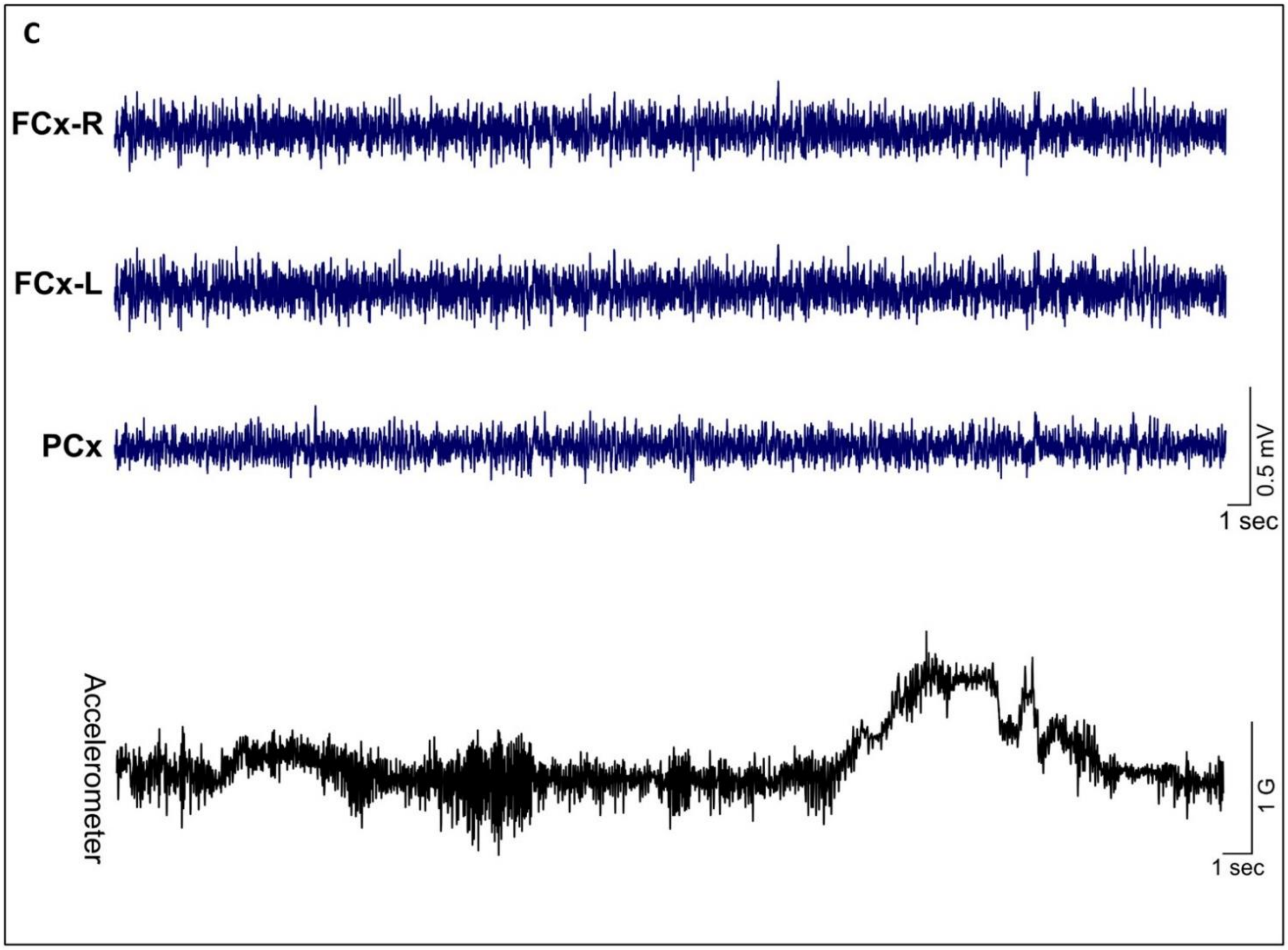

Supplementary Figure 7. Electroencephalogram (EEG) traces from normal WT FVB/N mice do not show any seizure activity – Example 3.

© 2023. Van Raamsdonk JM et al. Published online at http://www.aginganddisease.org/EN/10.14336/AD.2023.0423

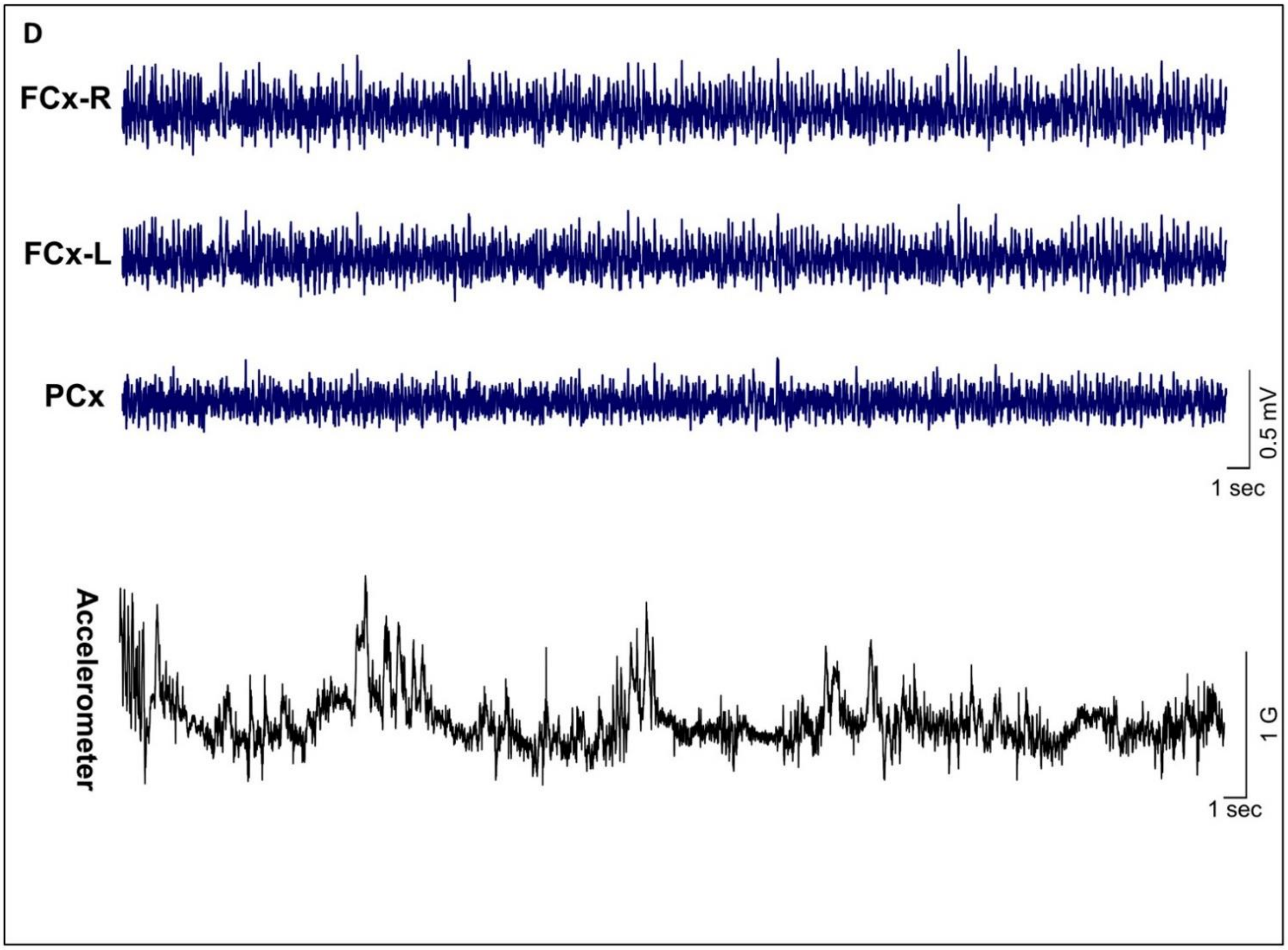

Supplementary Figure 8. Electroencephalogram (EEG) traces from normal WT FVB/N mice do not show any seizure activity – Example 4.

© 2023. Van Raamsdonk JM et al. Published online at http://www.aginganddisease.org/EN/10.14336/AD.2023.0423

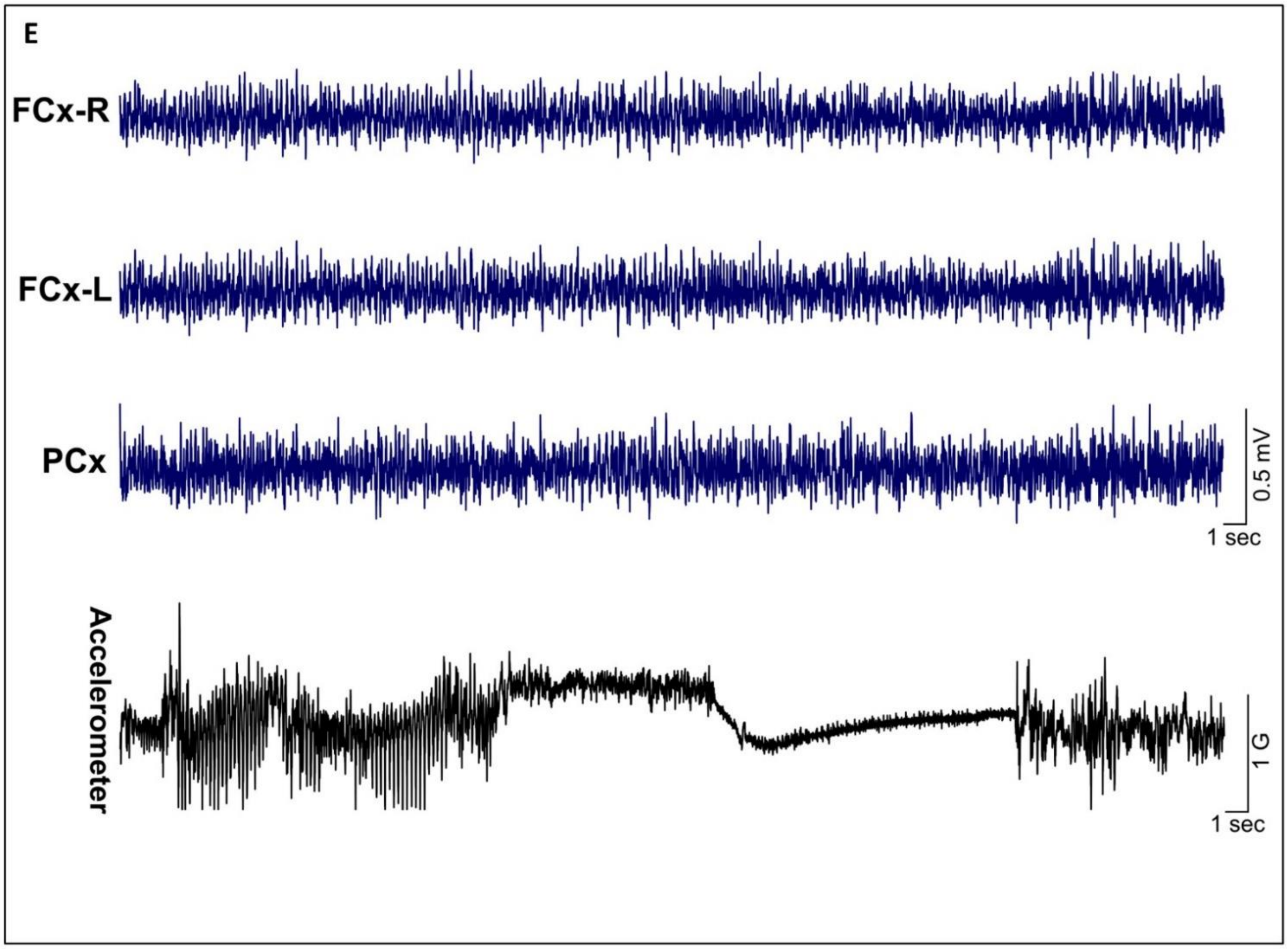

Supplementary Figure 9. Electroencephalogram (EEG) traces from normal WT FVB/N mice do not show any seizure activity – Example 5.

© 2023. Van Raamsdonk JM et al. Published online at http://www.aginganddisease.org/EN/10.14336/AD.2023.0423

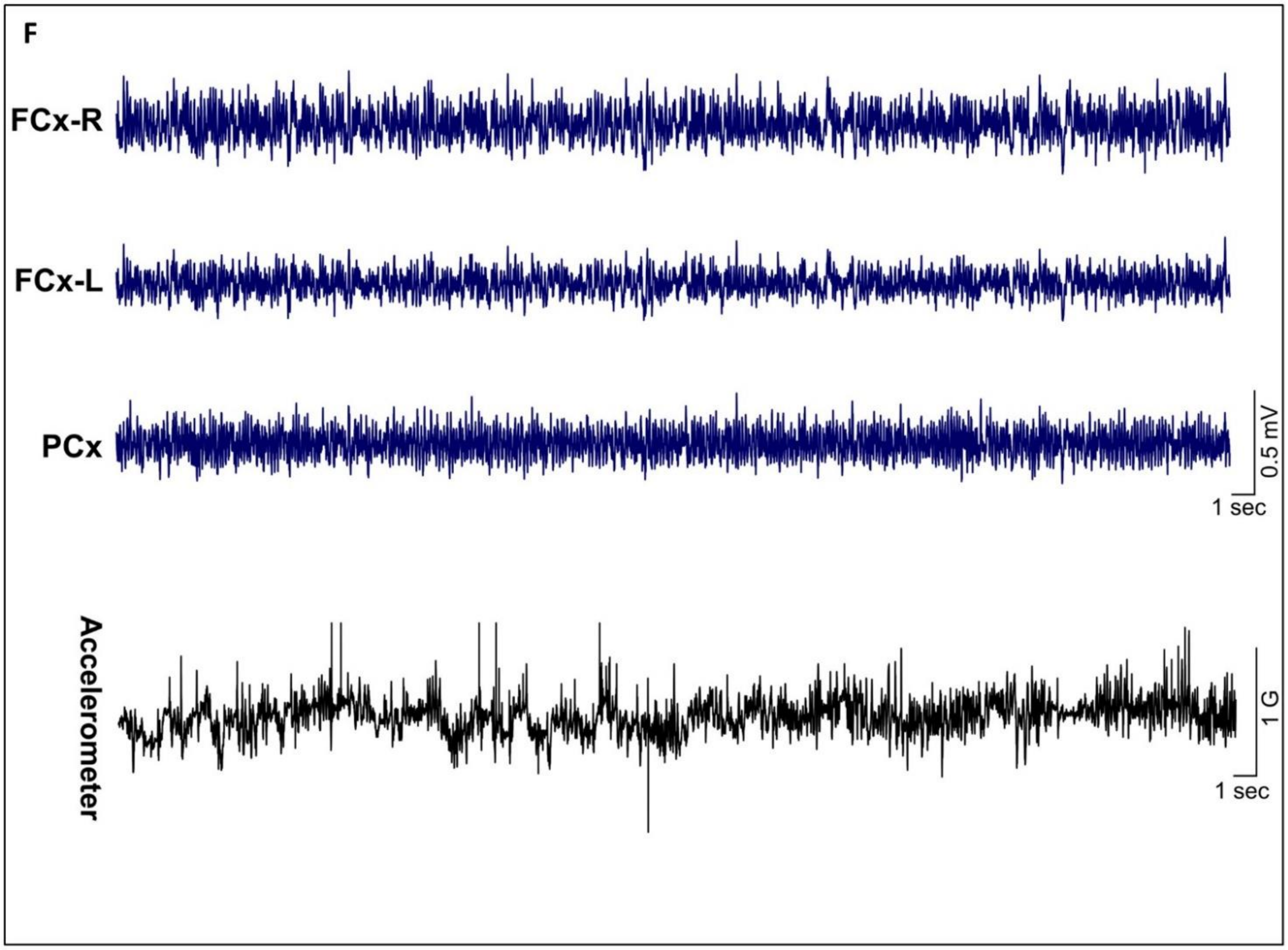

Supplementary Figure 10. Electroencephalogram (EEG) traces from normal WT FVB/N mice do not show any seizure activity – Example 6.

© 2023. Van Raamsdonk JM et al. Published online at http://www.aginganddisease.org/EN/10.14336/AD.2023.0423

SUPPLEMENTARY DATA

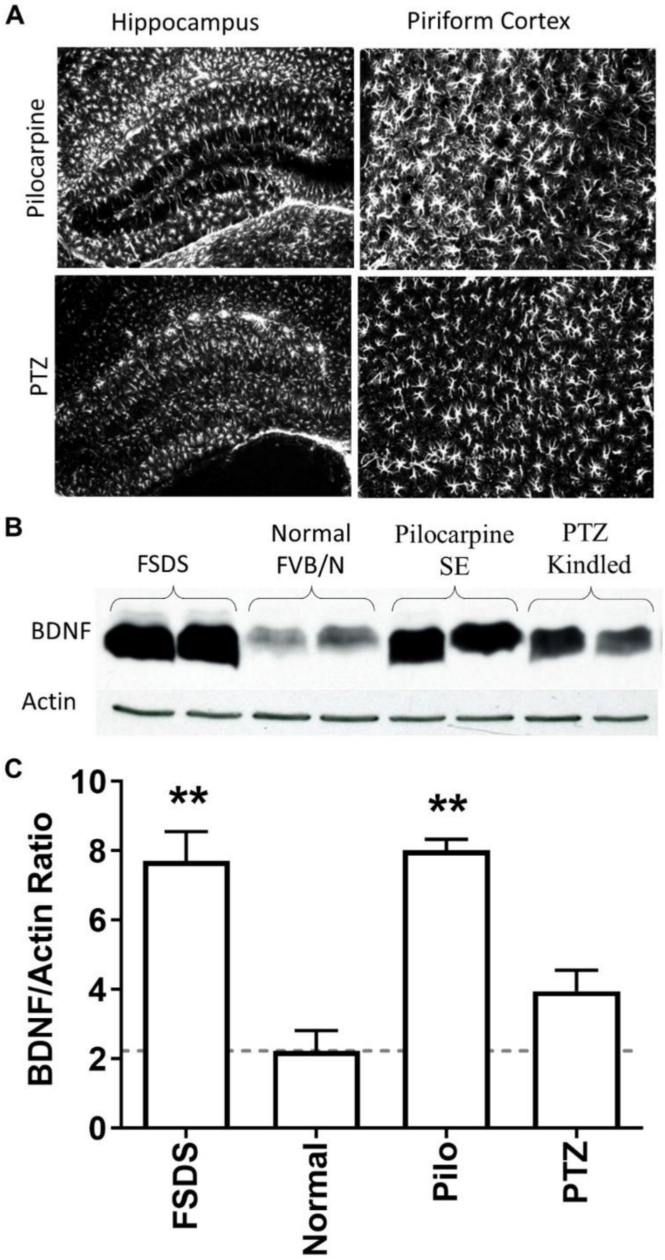

Supplementary Figure 11. Mouse models of epilepsy exhibit astrocytosis and upregulation of BDNF expression. In order to examine the seizure induced neurodegeneration in FSDS mice, we compared the brains of these mice with two established chemical models of epilepsy: kindling with penteylenetetrazole (PTZ) and pilocarpine induced status epilepticus. **(A)** As with FSDS mice, both pilocarpine and PTZ treated mice exhibited extensive astrocytosis in the hippocampus and piriform cortex. **(B)** Similarly, both of these chemical models of epilepsy showed increased levels of BDNF protein. **(C)** Quantification of BDNF levels showed that the increase in BDNF expression in FSDS mice was similar or greater to that in the two chemically induced seizure models (N=2 FSDS, N=2 Normal FVB/N, N=2 FVB/N mice pilocarpine-induced status epilepticus, N=3 FVB/N mice with PTZ kindling). Error bars indicates standard error of the mean. Statistical significance was assessed using a one-way ANOVA with Dunnett’s multiple comparisons test. ** $p < 0.01$.

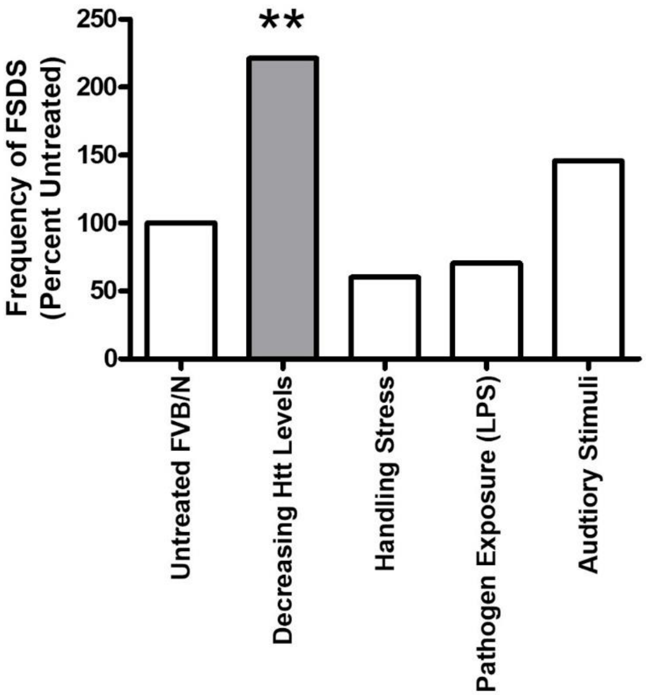

Supplementary Figure 12. FSDS incidence is unaltered by handling-stress, inflammation or auditory stimuli. Having shown that a decrease in Htt levels increased the frequency of the FSDS phenotype, we sought to determine whether the proportion of mice that develop this phenotype could be increased by manipulations that have previously been shown to influence the development of epilepsy in mouse or rat models. Unlike decreasing levels of Htt, the frequency of the FSDS phenotype was not altered by repeated handling, induction of inflammation with LPS (lipopolysaccharide) or auditory stimulation. Statistical significance was assessed using the Chi-squared test. ** $p<0.01$.

SUPPLEMENTARY DATA

Supplementary Table 1. Summary of statistical analyses.

Figure	Number of animals (N)	Statistical Test		Significance
1A	28 Normal FVB/N	N/A		
1B	31 FSDS	N/A		
1D	12 Normal FVB/N, 4 FSDS	two-way ANOVA with Šidák's multiple comparisons test	Total	p<0.0001
		D'Agostino-Pearson test for normality of residuals - passed	Forebrain	p<0.0001
			Cerebellum	p=0.9985
1F	6 Normal FVB/N, 5 FSDS	Mann-Whitney (non-parametric)		p=0.0043
2		N/A		
3C	3 Normal FVB/N, 3 FSDS	two-way ANOVA with Šidák's multiple comparisons test	Piriform cortex	p=0.0004
		D'Agostino-Pearson test for normality of residuals - passed	Amygdala	p<0.0001
			Striatum	p=0.8253
3E	3 Normal FVB/N, 3 FSDS	two-way ANOVA with Šidák's multiple comparisons test	Striatum	p<0.0001
		D'Agostino-Pearson test for normality of residuals - failed	Hippocampus	p<0.0001
			Cerebellum	p=0.9978
			Cortex	p<0.0001
3F	3 Normal FVB/N, 3 FSDS	two-way ANOVA with Šidák's multiple comparisons test	dmH3-K4	p=0.0016
		D'Agostino-Pearson test for normality of residuals - passed	acH4	p=0.0174
4B	17 WT, 14 Htt+/-	Chi square test		p=0.0015
4C	95 WT, 38 YAC18	Chi square test		p=0.0002
4D	56 WT, 56 YAC128	Chi square test		p<0.0001
4E	22 WT, 14 Shortstop	Chi square test		p=0.6
5	9 Normal FVB/N, 7 FSDS	Mann-Whitney (non-parametric)		p=0.7577
6D	6 WT, 4 YAC18	Mann-Whitney (non-parametric)		p=0.0381

SUPPLEMENTARY DATA

6A 7 WT saline, 17 WT PTZ, 19 YAC18 PTZ

Mixed-effects analysis
Tukey's multiple comparisons test

	1	3	5	8	10	12	15	17
WT - Saline vs. WT - PTZ	0.0003	0.0003	<0.0001	<0.0001	<0.0001	<0.0001	<0.0001	<0.0001
WT - Saline vs. YAC18 - PTZ	0.0001	<0.0001	<0.0001	<0.0001	<0.0001	<0.0001	<0.0001	<0.0001
WT - PTZ vs. YAC18 - PTZ	0.8687	0.906	0.9794	0.7167	0.9803	0.3904	0.5951	0.9371

6B 7 WT saline, 17 WT PTZ, 19 YAC18 PTZ

Mixed-effects analysis
Tukey's multiple comparisons test

	1	3	5	8	10	12	15	17
WT - Saline vs. WT - PTZ	0.1675	0.0704	0.0025	0.0024	0.0312	0.0023	0.0345	0.1676
WT - Saline vs. YAC18 - PTZ	0.2272	0.0402	0.0026	<0.0001	<0.0001	0.0069	0.0483	0.0388
WT - PTZ vs. YAC18 - PTZ	0.377	0.9108	0.6776	0.8895	0.6906	0.9744	0.5278	0.714

© 2023. Van Raamsdonk JM et al. Published online at http://www.aginganddisease.org/EN/10.14336/AD.2023.0423

SUPPLEMENTARY DATA

QQ Plots

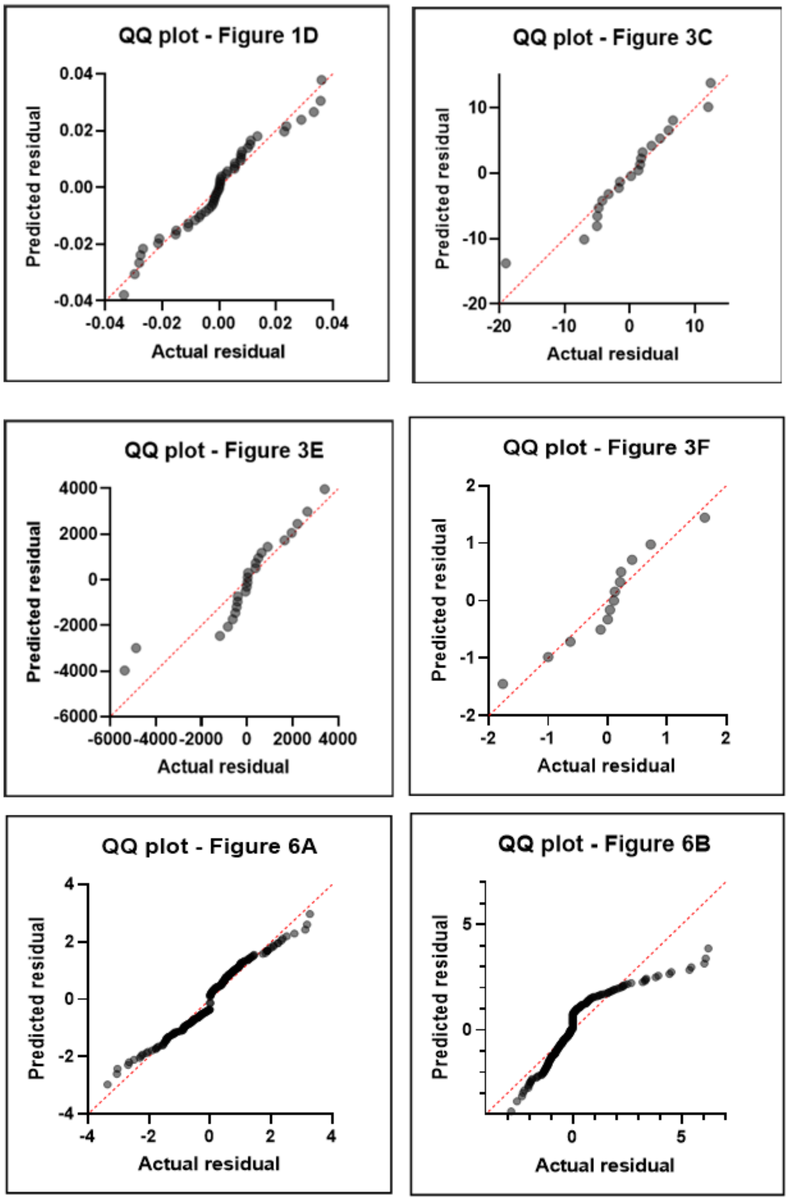

© 2023. Van Raamsdonk JM et al. Published online at http://www.aginganddisease.org/EN/10.14336/AD.2023.0423

SUPPLEMENTARY DATA

MOVIE LEGENDS

Movie 1. Home cage behavior of a normal FVB/N mouse. This movie illustrates the behavior of a normal FVB/N mouse. Notice that the mouse actively explores the cage.

Movie 2. Home cage behavior of an FSDS mouse. This movie illustrates the behavior of an FSDS mouse. In contrast to a normal FVB/N mouse, these mice remain immobile for long periods of time and do not explore their surroundings.

Movie 3. Home cage behavior of mouse following pilocarpine-induced *status epilepticus*. This movie illustrates the behavior of a mouse which has undergone status epilepticus following injection with pilocarpine. As with FSDS mice, these mice remain immobile for long periods of time and also do not explore their surroundings.

Movie 4. Spontaneous seizure observed in an FSDS mouse. This movie shows an FSDS mouse undergoing a spontaneous seizure. A normal FVB/N littermate walks past the seizing mouse.

Movie 5. Auditory SUDEP in an FSDS mouse. This movie shows an FSDS mouse undergoing SUDEP in response to an auditory stimulus. Before the auditory stimulus, the FSDS mouse is immobile. When the sound begins, the mouse responds by moving about the cage, first slowly then in a more frantic manner. Finally, the mouse exhibits a “popcorn” seizure and dies.

Movie 6. Comparison of normal FVB/N mouse and FSDS mouse in response to auditory stimulus. This movie compares the response of a normal FVB/N mouse and an FSDS mouse to an auditory stimulus. Before the sound begins, the normal FVB/N mouse is exploring the cage while the FSDS mouse is immobile. The auditory stimulus has no impact on the normal FVB/N mouse’s behavior. However, the auditory stimulus causes the FSDS mouse to move frantically about the cage, seize and then die.

© 2023. Van Raamsdonk JM et al. Published online at http://www.aginganddisease.org/EN/10.14336/AD.2023.0423
